# Surface Treatment of Composites with Bismaleimide Resin-Based Wet Peel Ply for Enhanced Adhesive Bonding Performance

**DOI:** 10.3390/polym13203488

**Published:** 2021-10-11

**Authors:** Hongfeng Li, Liwei Zhao, Yingjie Qiao, Xuefeng Bai, Dezhi Wang, Chunyan Qu, Changwei Liu, Yongqiang Wang

**Affiliations:** 1 Institute of Petrochemistry, Heilongjiang Academy of Sciences, Harbin 150040, China; tommybai@126.com (X.B.); jim603@163.com (D.W.); quchunyan168@163.com (C.Q.); ailp_liuchangwei@sina.com (C.L.); wangyongqiang376@163.com (Y.W.); 2 College of Material Science and Chemical Engineering, Harbin Engineering University, Harbin 150001, China; qiaoyingjie@hrbeu.edu.cn

**Keywords:** CFRP, wet peel ply, surface treatment, lap shear strength, failure mode

## Abstract

Surface treatment is typically required to improve the bonding performance of carbon-fiber-reinforced composites. Herein, a wet peel ply was prepared using bismaleimide (BMI) resins as a matrix resin. The temperature–heating rate extrapolation method and rheological method were employed to study the reaction characteristics and viscosity-temperature characteristics of the matrix in the BMI wet peel ply. The curing temperatures of the BMI wet peel ply and the BMI prepreg were the same (200 °C), making this wet peel ply suitable for co-curing with the BMI prepreg. After treatment with the wet peel ply, the bonding strength of the BMI composite joint showed a mean shear strength of 35.5 MPa, which was 1.72% higher than that of the sanded composite and 17.5% higher than that of the composite treated with the dry peel ply. In addition, the BMI composite treated with the BMI wet peel ply exhibited good bonding stability with a coefficient of variation of 3.9. After damp-heat aging for 1440 h, the retention rate of shear strength at room-temperature was 82.3%. The relatively loosely woven carrier in the BMI wet peel ply increased the surface roughness of the composite, thus improving the bonding strength.

## 1. Introduction

Carbon-fiber-reinforced polymer (CFRP) composites, which have better specific strength [1], specific [2], corrosion resistance [3], and fatigue resistance [4] than other materials, are increasingly used in the aviation industry [5,6,7]. The connection technology of CFRPs mainly includes bonding connection [8] and mechanical connection [9,10]. The mechanical connection can transmit large loads, but the holes on the CFRPs will cause serious stress concentration [11], which will weaken the strength of the material. The bonding method will not cause damage to the CFRP material. The bonding also has the advantages of excellent fatigue resistance, high connection efficiency, and large load transfer. It has become the main connection technology for CFRP materials in the aerospace field [12]. In the aerospace industry, the bonding of CFRPs includes co-curing, co-bonding, and secondary bonding [13]. The CFRP laminate is used in co-bonding and secondary bonding [14,15,16,17], so the surface treatment has an important influence on the bonding performance [18,19].

Debris and contaminants on the laminate surface must be removed without the use of detergents. A surface suitable for bonding can be obtained using abrasives, grit blasting, peel ply, tear ply, electric corona discharge, plasma treatment, and flame treatment and laser ablation [20]. For example, Tao [21] et al. showed that pulsed CO_2_ laser irradiation effectively enhanced the mode I fracture toughness of adhesively bonded composite joints. Law [22] et al. found that composites treated using air-based plasma instead of grit blasting prior to composite-to-composite adhesive bonding exhibited a 7% enhancement in lap-shear strength. Dehaghani [23] et al. used acid etching to treat the surface of a polyethylene liner and investigated the effect of surface treatment duration on the adhesive bonding of E-glass/epoxy composites to the liner. However, abrasives [24] or peel plies [25,26] are generally used for surface treatment in the actual manufacturing process. Mechanical abrading is a labor-intensive, worker’s-skill-dependent, costly phase [27], and the treatment quality is difficult to measure. Since peel plies can protect the surface during transport and storing the parts and can produce a clean, chemically active surface with the required roughness for bonding when they are removed from the composite, they are generally used as part of the composite manufacturing process in the aerospace industry [28]. Dry peel ply is an extra layer of fabric material, and it absorbs some of the matrix resin during the cure fabrication process [29]. It will damage the fiber layer of the composite material when dry peel ply is peeled off. A wet peel ply contains fabric and a resin system similar to the matrix resin of the composite material, so it is beneficial to protect the composite layer. Buchmann [25] et al. studied the effect of peeling angle, peel ply orientation, and peel ply material on the peeling force required to remove the peeling layer from the CFRP surface, and the results showed that a sharp peel angle close to 180° could reduce the peeling force and fiber breakouts. Martínez-Landeros [30] et al. compared the effects of different surface treatment types on the bonding effect of structural adhesives for CFRP. The results showed that peel ply exhibits more stable load-displacement data and G_IC_ values with reduced variability. Various studies have shown that wet peel plies are not universally applicable but show selectivity for composite materials and structural adhesive films [31]. Therefore, the composite, wet peel ply and adhesive must be selected as a system [32].

Despite the widespread use of this process, bonding strength and durability can be improved by preparing wet peel ply that matches CFRP [33]. The industry and academia continue to study the factors that affect the adhesion performance of the composite material/release layer/adhesive system and the development of other new material systems. Therefore, in this study, a bismaleimide (BMI)-based wet peel ply was prepared for co-bonding of a novel heat-resistant BMI composite. A modified BMI resin system that is similar to the BMI composite matrix resin system was designed for the wet peel ply. The matrix resin of the wet peel ply can be cured according to the curing process of the BMI prepreg, and the cured matrix exhibited excellent thermal stability, with a glass transition temperature of 200 °C. Adhesively bonded joints of BMI composite treated by the fabricated wet peel ply showed excellent shear strength and bonding stability compared to that of sanding and with a dry peel ply. Furthermore, the BMI composite treated with the wet peel ply showed excellent shear strength retention after damp-heat aging for 1440 h.

## 2. Materials and Methods

### 2.1. Materials

J-412 BMI-based wet peel ply and J-188 BMI film adhesive were obtained from the Institute of Petrochemistry, Heilongjiang Academy of Sciences (Harbin, China). BMI-700 resin-based carbon fiber prepreg was obtained from the China Aerospace Research Institute of Materials & Processing Technology (Beijing, China), and Release Ply G dry peel ply was obtained from Airtech (Huntington Beach, CA, USA).

### 2.2. Sample Preparation

#### 2.2.1. Preparation of BMI Composite Laminates

The preparation of the composite laminate was in accordance with the requirements of ASTM5687. Sixteen layers of the-BMI-700 prepreg in a unidirectional tape were laid in the 0° direction to fabricate the BMI laminates. Three kinds of composite laminates were prepared: a composite laminate directly cured with no peel ply on the surface, a composite laminate co-cured with the J-412 BMI wet peel ply on the surface, and a composite laminate cured with the Release Ply G dry peel ply on the surface. The samples were cured in an autoclave under the following conditions given by the prepreg supplier: the sample was cured at a constant temperature of 200 °C for 3 h with a vacuum of 0.1 MPa and an external pressure of 0.5 MPa.

#### 2.2.2. Fabrication of Double-Lap Joints

For the laminates with peel plies on the surface, the peel plies were removed before bonding, whereas the laminate with no peel ply on the surface was manually sanded. The laminates were co-cured and bonded with the J-188 BMI film adhesive to 8 layers of the BMI prepreg laid in the 0° direction. The curing process was performed in an autoclave under the following conditions: the sample was cured at a constant temperature of 200 °C for 3 h with a vacuum of 0.1 MPa and an external pressure of 0.5 MPa.

**Figure 1:**
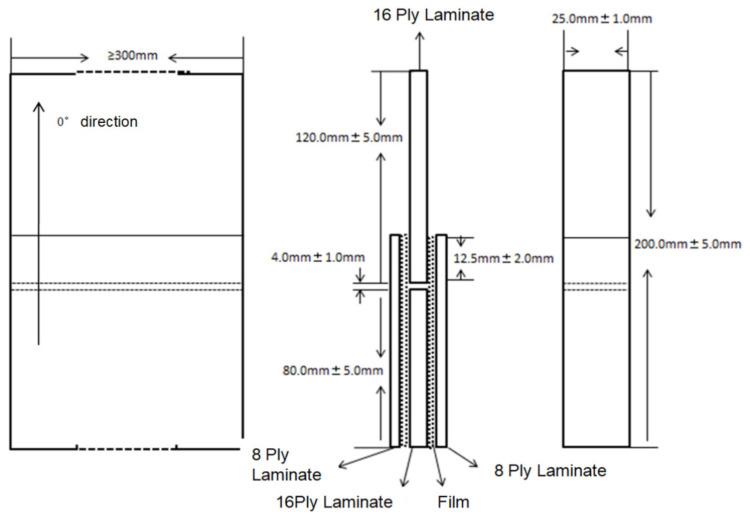
Schematic of a composite sample for double-lap shear testing.

The specifications, size, bonding form, and bonding area of the double-lap shear testing samples are shown in Figure 1. The digital images of double-lap shear test samples are shown in Figure S1.

### 2.3. Characterization

To investigate the thermal properties of the composites, differential scanning calorimetry (DSC) was conducted using a differential scanning calorimeter (Q20, TA Instruments, New Castle, DE, USA) in the temperature range of 25–350 °C at heating rates of 5, 10, 15, and 20 °C/min (nitrogen atmosphere). The static and dynamic viscosity and processing temperature characteristics were evaluated using a rheometer (DHR-1, TA Instruments, Linton, UT, USA). The double-lap shear strength was tested according to ASTM D3528 using an Instron 4505 electronic materials testing machine at a test rate of 1.25 ± 0.25 mm/min. Scanning electron microscopy (SEM) was performed using a scanning electron microscope (JSM-IT300, JEOL, Tokyo, Japan) to observe the surface morphologies of the samples. The fractured surfaces were sputter-coated with gold in an argon environment using an SC7620 sputter coater. Three-dimensional microscopy was performed using an RH-2000 video microscope to obtain the images. The dynamic mechanical analysis (DMA) of the specimen was tested on a dynamic mechanical analyzer (Q800, TA Instruments, New Castle, DE, USA). The shape of the specimen was 50 mm × 10 mm × 3 mm. The test was run in single cantilever mode with a frequency of 1 Hz and amplitude of 15 µm. The temperature rose from room temperature to 240 °C at a rate of 5 °C/min.

## 3. Results and Discussion

### 3.1. Rheological Analysis

**Figure 2:**
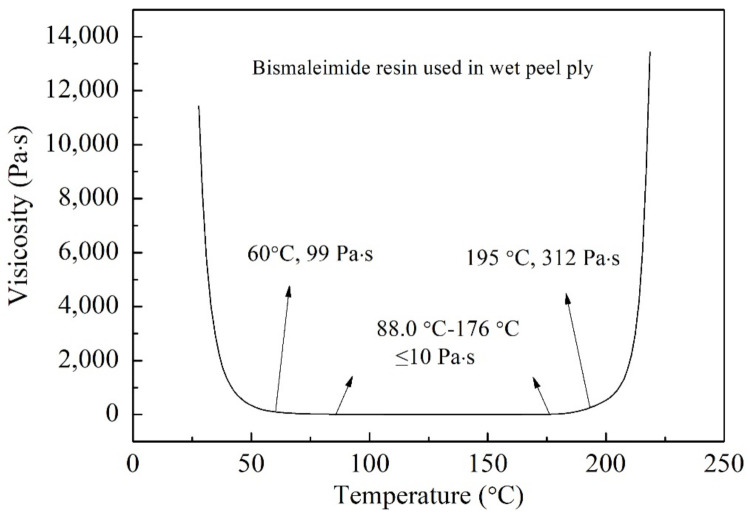
Viscosity–temperature curve of the matrix resin of the wet peel ply.

Figure 2 shows the viscosity–temperature curve of the matrix resin of the J-412 BMI-based wet peel ply. The viscosity of the matrix resin decreased continuously as the temperature increased up to 50 °C, whereas the viscosity changed insignificantly with temperature when the temperature was above 60 °C, indicating that that the viscosity of the resin before curing is temperature-dependent and that the temperature is a critical parameter in the manufacturing process of wet peel plies. The lowest viscosity (≤10 Pa·s) was observed at temperatures between 88 °C and 176 °C, indicating that the J-412 wet peel ply has a wide curing temperature window. Gelation of the matrix occurred at temperatures above 176 °C, resulting in increase in the viscosity. The viscosity increased rapidly above 195 °C when gelation and the curing reaction intensified. This viscosity–temperature curve can serve as a reference for developing co-curing processes for peel plies and prepregs.

### 3.2. Analysis of Curing Reaction Process

**Figure 3:**
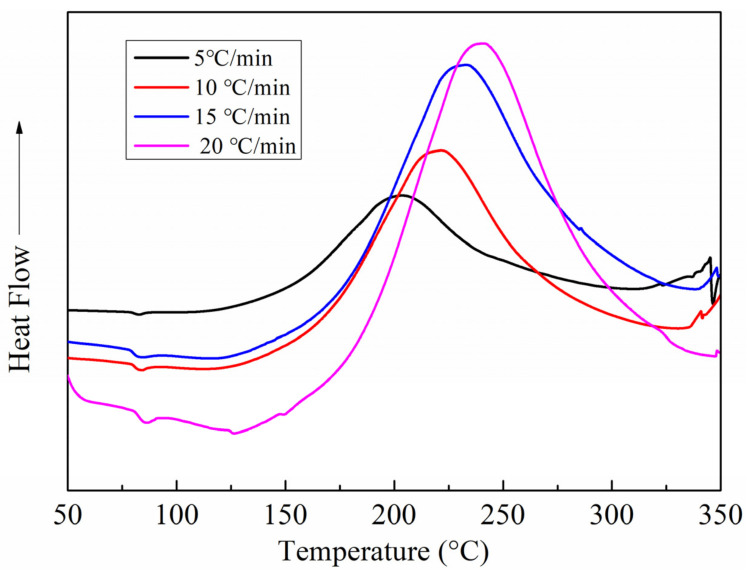
Differential scanning calorimetry (DSC) curves of the matrix resin of the wet peel ply.

Figure 3 shows the DSC curves of the matrix resin of J-412 BMI wet peel ply at different heating rates (5, 10, 15, and 20 °C/min). As shown in Table 1, the characteristic temperatures of the matrix resin (initial temperature (Ti), peak temperature (Tp), and final temperature (Tf)) increased with the increase in heating rate. In addition, it can be seen from Figure S2 that the reaction enthalpy ΔH was in the range of 309.2–348.0 J/g, indicating that the increase in the heating rate had little effect on the curing reaction enthalpy.

**Figure 4:**
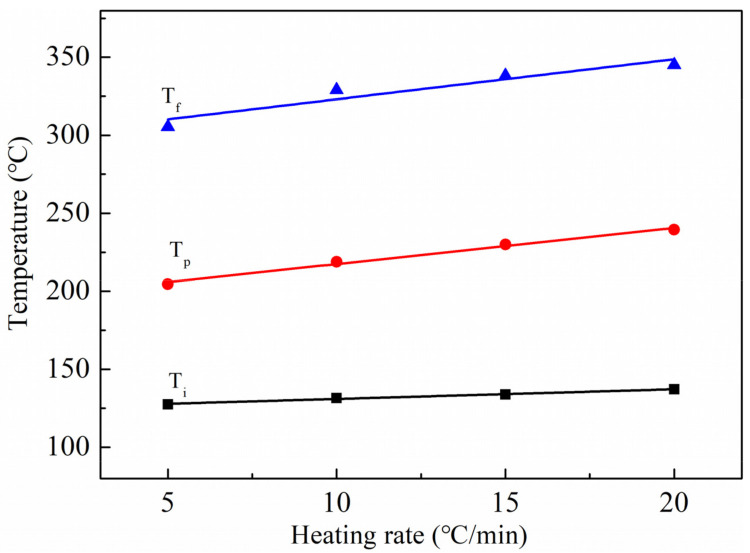
Linear fitting of initial temperature (T_i_), peak temperature (T_p_), and final temperature (T_f_) vs. heating rate (β) plots.

The temperature–heating-rate extrapolation method was employed to determine the curing temperatures of the matrix resin. The characteristic temperatures (T_i_, T_p_, and T_f_) were plotted against the heating rate (β), and linear regression was performed, as shown in Figure 4. Extrapolation of the T_i_–β, T_p_–β, and T_f_–β curves to a heating rate of zero gave the gelation, curing, and post-treatment temperatures, respectively, which are characteristic of the curing process. The gelation, curing, and post-treatment temperatures were 125 °C, 195 °C, and 297 °C, respectively. The curing temperature of the wet peel ply matched that of the BMI prepreg (200 ± 5 °C), thus the curing process of the matrix resin of wet peel ply satisfies the requirement for co-curing with BMI prepreg. 

### 3.3. Thermal Stability and Heat Resistance

**Figure 5:**
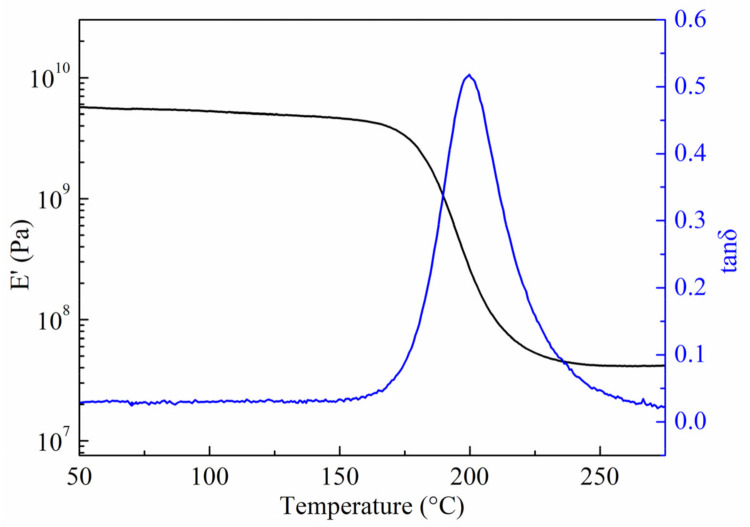
Storage modulus (E’) and tanδ of the matrix resin obtained from DMA method.

The BMI resin used in the wet peel ply also exhibited excellent thermal stability and heat resistance properties. The glass transition temperature (Tg) and thermal decomposition temperature can be used to study the compatibility of the composites/adhesive/peel ply system in heat resistance. Since some resin in the peel ply will be left on the bonding surface of the composites when the peel ply is removed, excellent heat resistance is the guarantee for the joint to maintain outstanding bonding strength at high temperatures. In order to study the thermal stability of BMI resin used in wet peel ply, the storage modulus and tanδ of matrix were obtained, as shown in Figure 5. It can be seen that the matrix resin exhibited a higher storage modulus in the glass state. In addition, the Tg of matrix resin reached 200 °C, so the bonded composite joint can exhibits higher bonding strength at temperatures up to 150 °C, improving the stability of the joints at high temperatures. 

**Figure 6:**
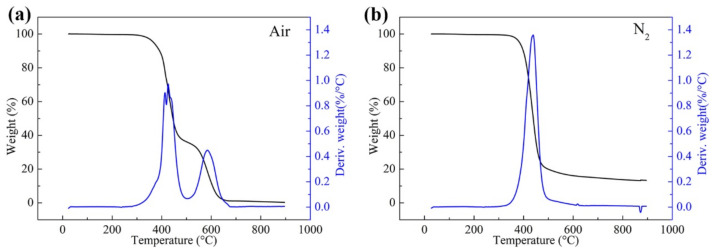
TG and DTG curves of the matrix resin in (**a**) air and (**b**) nitrogen atmospheres.

The thermal weight losses in air and nitrogen atmospheres are shown in Figure 6. It can be seen that the BMI resin had almost no residue due to the combustion of carbon in the air. The residual amount of BMI resin in N_2_ was about 15%. The thermal weight loss of the resin system in air can be divided into two stages, which is attributable to the oxidative degradation and combustion of carbonaceous residues, respectively. The temperature at 5% weight loss was 367.1 °C, and the temperature corresponding to the maximum degradation rate was 425.3 °C. The temperature at 5% weight loss under nitrogen was 383.7 °C, and the temperature corresponding to the maximum degradation rate was 444.5 °C. These results show that the matrix resin system exhibits excellent heat resistance enough to be used in wet peel ply for BMI-700 composite.

### 3.4. Microstructure Analysis of Different Laminate Surface

**Figure 7:**
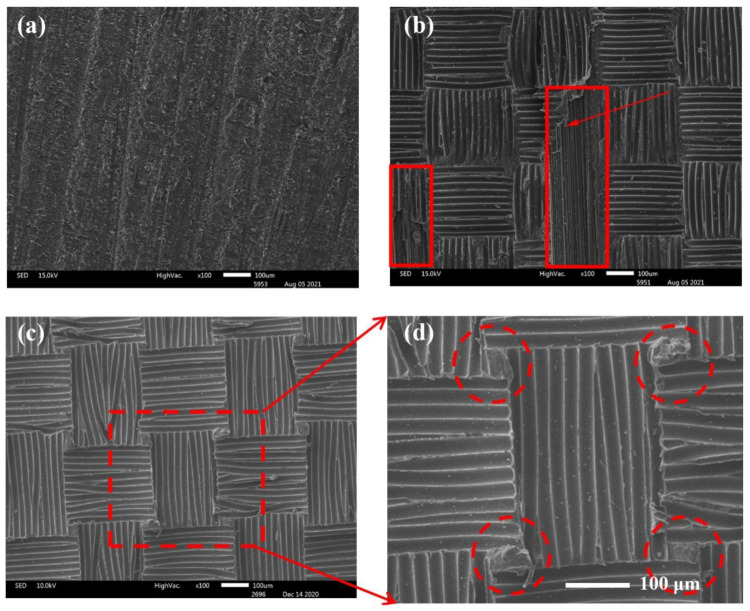
SEM images of composite surfaces treated using different methods: (**a**) sanding, (**b**) dry peel ply, and (**c**,**d**) wet peel ply.

SEM was used to study the microstructure of the composite surfaces after different surface treatments, as shown in Figure 7. Sandpaper sanding removed a thin layer of resin on the surface of the composite and created unidirectional grooves (Figure 7a). This method increased the roughness of the bonding surface, which is beneficial for improving the bonding strength. However, this mechanical method is likely to cause damage to the carbon fibers in the composite. Dry peel ply can also improve the surface roughness of the composite material and create a fresh surface for bonding. However, since the dry cloth absorbed the resin during the co-curing process with the prepreg, the carbon fiber layer was destroyed when it was peeled off, as shown in Figure 7b. In contrast, the composite treated with the wet peel ply showed a regular resin texture on the surface, and the surface roughness of the composite was improved without damaging the carbon fiber layer (Figure 7c,d).

**Figure 8:**
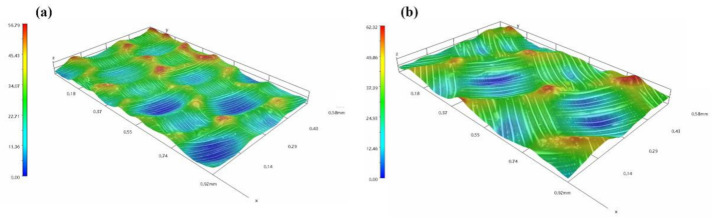
Three-dimensional images of the composite surface (**a**) after removing of the wet peel ply (**b**).

Three-dimensional microscope images of the composite surface treated with the wet peel ply are shown in Figure 8a,b. The surface of the composite was neat and free of obvious residual debris or peel ply fabric. The surface of the composite became uneven after removing the cured wet peel ply, with distinct weaving marks that replicated the texture of the carrier. In addition, there was residual resin that remained at the interlaced areas of the woven texture, which was observed as protrusions. When the peel ply was peeled off, the fabric was peeled off along with the matrix resin. Matrix resin bonds that connect the upper and lower resin layers through gaps in the warp and weft yarns were simultaneously broken. The results show that as the peel ply was loosely woven, there were gaps between the warp and weft yarns, which resulted in resin-rich areas on the composite surface. Such features could increase the surface roughness, which increased mechanical occlusion and improved the bonding strength.

### 3.5. Bonding Performance and Failure Modes of Composites Treated Using Different Methods

The bonding performance of the composites with different surface treatments was evaluated using the shear strength. Double-lap shear testing was performed to avoid interlaminar failure caused by shear lag, bending, and the end effects of bonded joints. The composite was bonded using the J-188 BMI film adhesive after surface treatment (sanding, dry peel ply, or wet peel ply). The double-lap shear strength was obtained at room temperature (23 °C), and the test results are shown in Table 2. The images of samples used for double-lap shear testing samples before and after the tests are shown in Figure S3.

The composite treated by sanding exhibited a mean double-lap shear strength of 34.9 MPa and maximum shear strength of 39.1 MPa, which was the highest among all the measured samples. However, the minimum strength of 30.4 MPa was also the lowest among all measured samples, resulting in a high coefficient of variation (CV) of 10.8. These results indicate that sanding offers good bonding performance, but huge variance occurs, which can be attributed to uncertainty in the manual operation. Compared with the other two treatment methods, the composite treated with the dry peel ply showed slightly low bonding strength, probably because the surface structure of the composite was destroyed when the dry peel ply was torn off, removing some matrix resin from the composite and damaging the surface fibers. The composite treated with the J-412 BMI wet peel ply showed a mean shear strength of 35.5 MPa, which was 1.72% than that of the sanded composite and 17.5% higher than that of the composite treated with the dry peel ply. Moreover, for the samples treated with the wet peel ply, the measured double-lap shear strength showed the least variation, with a CV of 3.9, indicating high bonding stability. 

**Figure 9:**
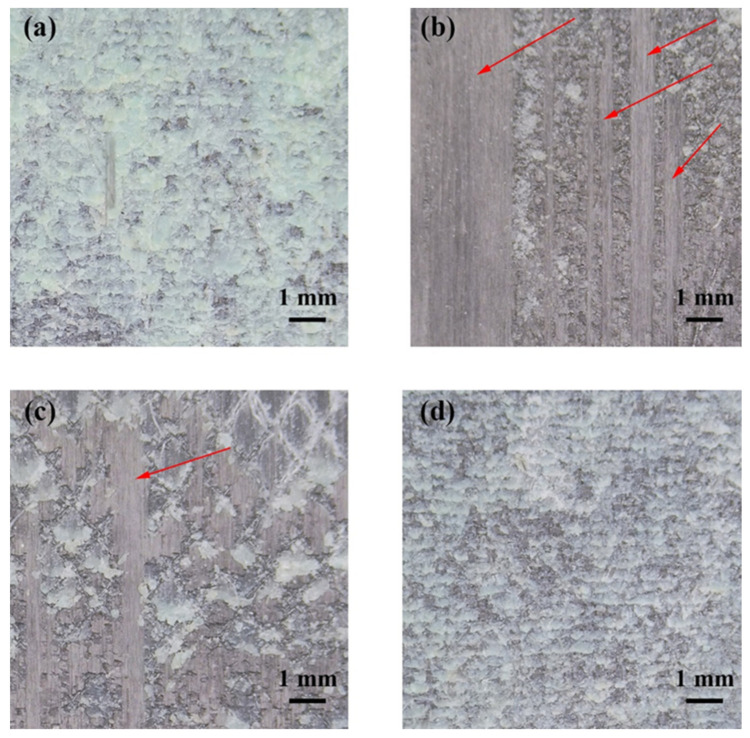
Failure modes of shear test samples with different surface treatment: (**a**,**b**) sanding, (**c**) dry peel ply, and (**d**) wet peel ply.

For adhesive-bonded composite joints, both the failure mode and the shear strength of the joint are important for evaluating bonding performance. Figure 9 shows the optical photographs of composite joints after the test under different surface treatment methods. Among the sanded samples, the failure modes include cohesive failure of the adhesive (Figure 9a) and interlayer failure of composite materials (Figure 9b). The failure modes of the shear test samples treated with dry and wet peel plies are shown in Figure 9c,d, respectively. The shear test samples treated with either that dry or wet peel ply mainly exhibited cohesive failure. However, the composite treated with the dry peel ply showed interlayer failure of the composite. These results indicate that the composite treated with the wet peel ply exhibited both excellent bonding performance and good bonding stability.

### 3.6. Comprehensive Bonding Performance of Composite Joints Treated with the Wet Peel Ply

**Figure 10:**
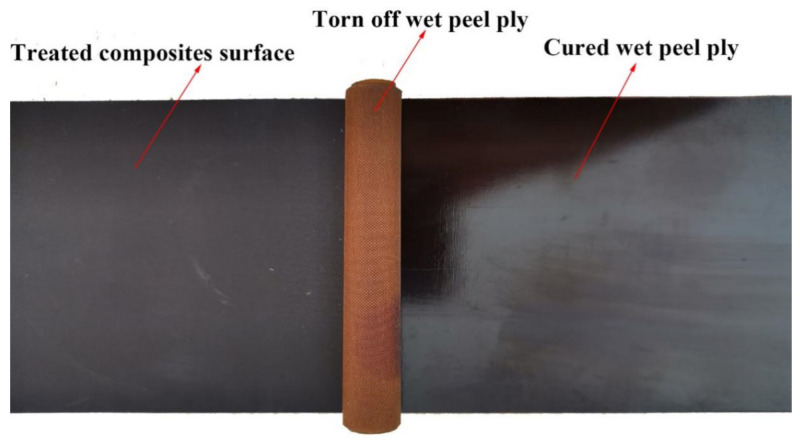
Cured BMI laminate with the wet peel ply.

The BMI-700 composite treated with the J-412 BMI wet peel ply was bonded to form double-lap shear test samples using the J-188 BMI film adhesive. A cured BMI laminate with the wet peel ply is shown in Figure 10. The cured wet peel ply can ensure the surface quality of the composite laminate and can also protect the composite from physical scratches, and this is advantageous during the transportation and storage of composite materials.

**Figure 11:**
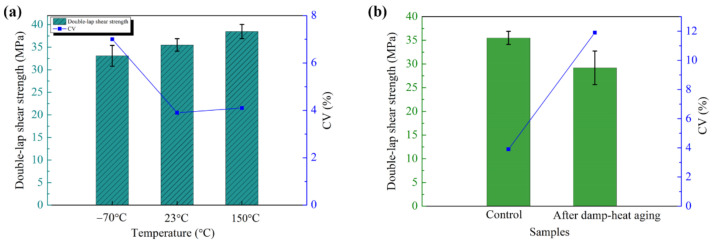
(**a**) Bonding performance of BMI-700 carbon fiber composite treated with J-412 BMI wet peel ply and (**b**) double-lap shear strength upon damp-heat aging (RH 95–100%, 71 ± 2 °C, 1440 h).

The comprehensive performance of the composite treated with the wet peel ply was evaluated, and the results are shown in Figure 11. The mean double-lap shear strength at room temperature reached 35.5 MPa, implying superior bonding performance. The CV of 3.9 indicated a small deviation and good bonding stability. The double-lap shear strength remained high at −70 °C and 150 °C (Figure 11a), indicating that the composite joint exhibited high resistance to both high and low temperatures. In particular, the shear strength at 150 °C was 3.0 MPa higher than that at room temperature, confirming the excellent heat resistance of the composite/BMI film adhesive/wet peel ply system. After 1440 h of damp-heat aging, the double-lap shear strength decreased but remained high (Figure 11b), with a shear strength of 29.2 MPa at room temperature, corresponding to a performance retention rate of 82.3%. Thus, the BMI-700 composite/J-188 BMI film adhesive/J-412 BMI wet peel ply system had good resistance against damp-heat aging.

## 4. Conclusions

To improve the co-bonding strength and bonding stability of BMI-700 CFRP and to provide protection for this CFRP, the J-412 BMI wet peel ply was designed and prepared by using a matrix resin similar to that of the BMI-700 prepreg. The temperature window for curing the J-412 BMI wet peel ply exhibited low viscosity in a wide temperature window of 60–175 °C, which is suitable for the manufacture of composite materials. The curing temperature of the wet peel ply matched that of the BMI-700 prepreg, satisfying the requirement for co-curing with the BMI prepreg. The loose fabric and the matrix resin in the J-412 BMI wet peel ply increased the surface roughness of the composite, resulting in improved bonding strength and bonding stability. Moreover, it can also provide protection for composite parts from physical damage. The composite treated with the J-412 BMI wet peel ply showed a mean shear strength of 35.5 MPa, which was 1.72% higher than that of the sanded composite and 17.5% higher than that of the composite treated with the dry peel ply. Moreover, the samples treated with the wet peel ply showed the least variation, with a CV of 3.9, indicating high bonding stability. After damp-heat aging for 1440 h, the double-lap shear strength at room temperature was 29.2 MPa, corresponding to a retention rate of 82.3%. This demonstrates that the bonded joint of the composite treated with the wet peel ply had excellent resistance to dampness and heat. Although this wet peel ply can simultaneously provide protection for the BMI-700 composite material and improve the bonding strength and bonding stability of the bonded joint, the composites/adhesive/wet peel ply should be selected as a system. The designed BMI wet peel ply is very suitable for composite/adhesive system with similar matrix resin systems. The methodology proposed in this work can provide a reference for the design and application of other wet peel ply for the composite/adhesive/wet peel ply system.

## Figures and Tables

**Table 1:** Characteristic temperatures of the matrix resin obtained from the DSC curves at different heating rates.

Heating Rate (°C/min)	Initial Temperature, T_i_ (°C)	Peak Temperature, T_p_ (°C)	Final Temperature, T_f_ (°C)
5	127.6	204.6	305.4
10	131.6	218.8	329.1
15	133.9	230.0	338.3
20	137.2	239.4	345.1

**Table 2:** Bonding performance of composite joints.

Scheme	Double-Lap Shear Strength (MPa)	Mean Shear Strength (MPa)	Standard Deviation	Coefficient of Variation, CV (%)
Sanding	38.4, 32.5, 34.2, 30.4, 39.1	34.9	3.75	10.8
Dry peel ply	32.1, 31.5, 29.6, 28.8, 29.0	30.2	1.50	5.0
Wet peel ply	37.5, 35.9, 35.1, 33.7, 35.3	35.5	1.38	3.9

## Data Availability

The data presented in this study are available on request from the corresponding author.

## Supplementary Materials

The following are available online at https://www.mdpi.com/article/10.3390/polym13203488/s1, Figure S1: Digital images of double lap shear test samples, Figure S2: The integrated DSC curves of the matrix resin of the wet peel ply at different heating rates, Figure S3: Comparison images of double lap shear samples before and after testing.
