# Supplementary material for: Surface Treatment of Composites with Bismaleimide Resin-Based Wet Peel Ply for Enhanced Adhesive Bonding Performance
Source: Polymers (Basel). 2021 Oct 11;13(20):3488. doi: 10.3390/polym13203488 (PMC8538825; doi:10.3390/polym13203488)
Supplement: Supplementary file 1 [file polymers-13-03488-s001.zip › polymers-1380244-supplementary.pdf]

Surface Treatment of Composites with Bismaleimide Resin-Based Wet Peel Ply for Enhanced Adhesive Bonding Performance

Hongfeng Li[1, 2,*], Liwei Zhao[1, 2,*], Yingjie Qiao[2], Xuefeng Bai[1], Dezhi Wang[1], Chunyan Qu[1], Changwei Liu[1], Yongqiang Wang[1]

[1] Institute of Petrochemistry, Heilongjiang Academy of Sciences, Harbin 150040, China; tommybai@126.com (X.B.); jim603@163.com (D.W.); quchunyan168@163.com (C.Q.); ailp_liuchangwei@sina.com (C.L.); wangyongqiang376@163.com (Y.W.)

[2] College of Material Science and Chemical Engineering, Harbin Engineering University, Harbin 150001, China; qiaoyingjie@hrbeu.edu.cn

* Correspondence: lihongfengcn@126.com (H.L.); zhaoliwei0130@163.com (L.Z.)

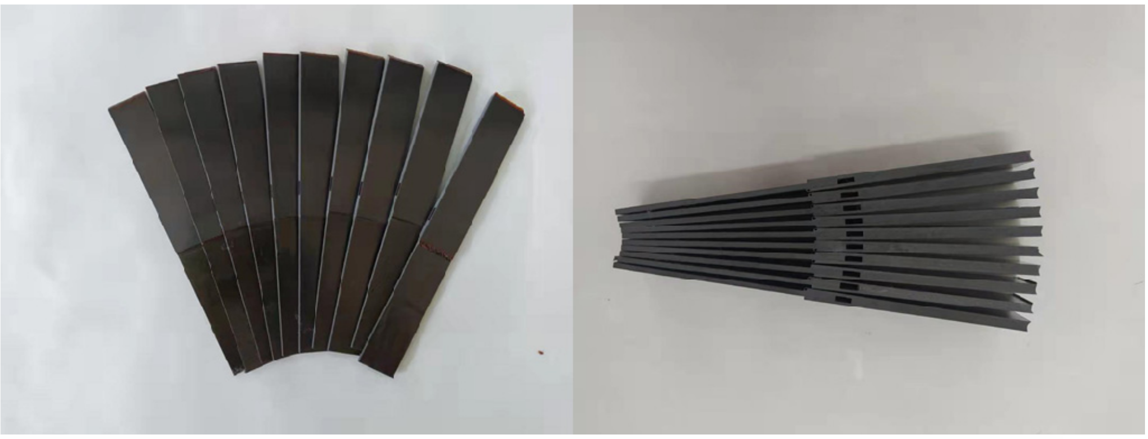

Figure S1. Digital images of double lap shear test samples.

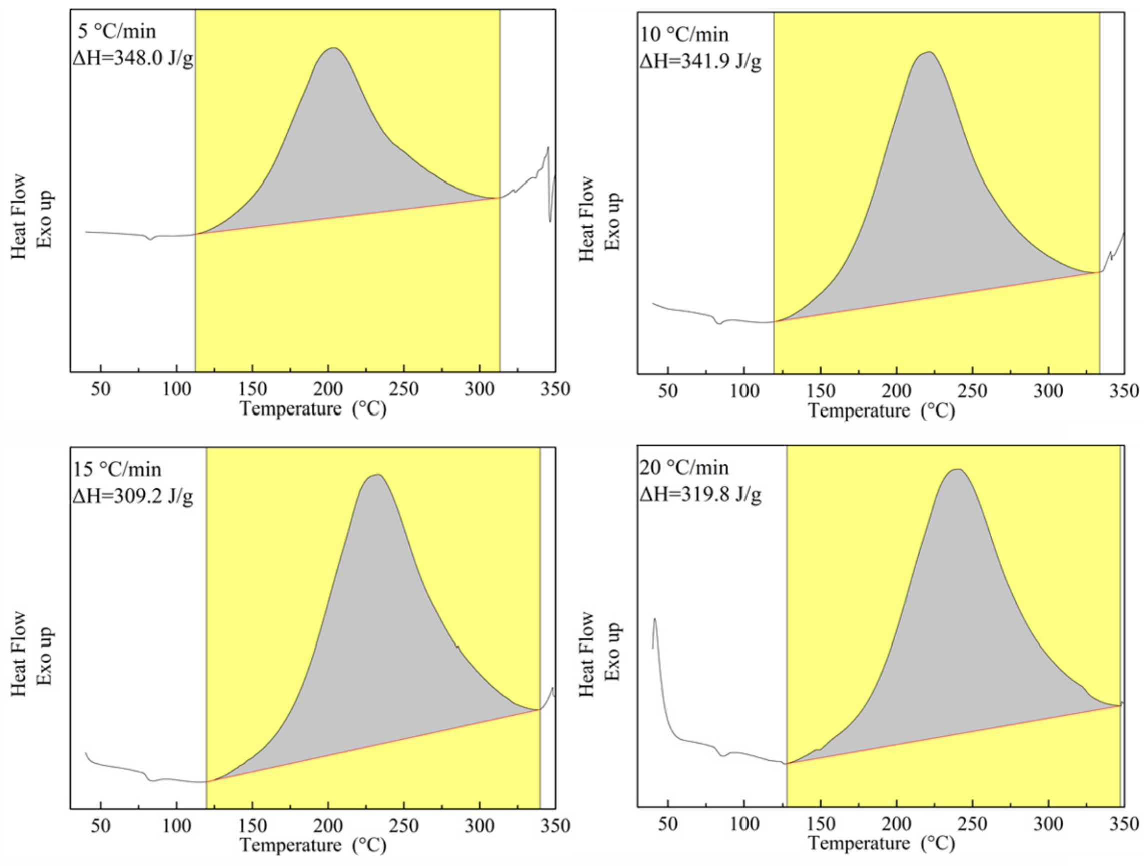

Figure S2. The integrated DSC curves of the matrix resin of the wet peel ply at different heating rates.

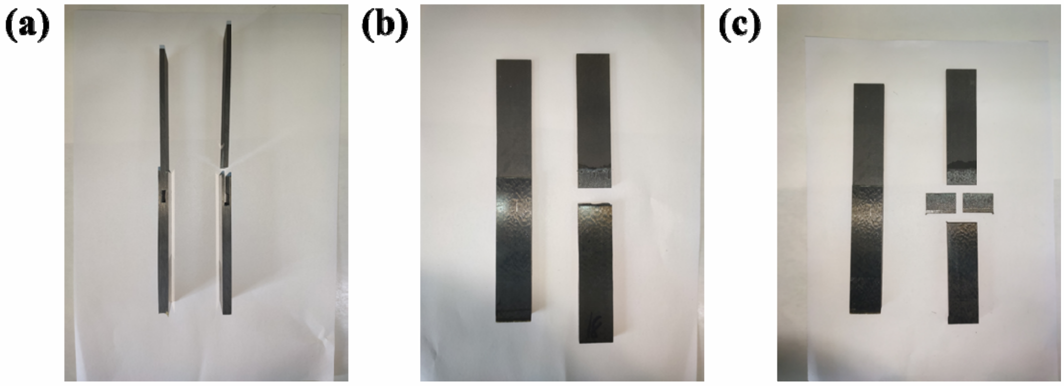

Figure S3. Comparison images of double lap shear samples before and after testing.
